# Prehypertension-Associated Elevation in Circulating Lysophosphatidlycholines, Lp-PLA_2_ Activity, and Oxidative Stress

**DOI:** 10.1371/journal.pone.0096735

**Published:** 2014-05-06

**Authors:** Minjoo Kim, Saem Jung, Su Yeon Kim, Sang-Hyun Lee, Jong Ho Lee

**Affiliations:** 1 National Leading Research Laboratory of Clinical Nutrigenetics/Nutrigenomics, Department of Food and Nutrition, College of Human Ecology, Yonsei University, Seoul, Korea; 2 Department of Food and Nutrition, Brain Korea 21 PLUS Project, College of Human Ecology, Yonsei University, Seoul, Korea; 3 Interdisciplinary Course of Science for Aging, Yonsei University, Seoul, Korea; 4 Department of Family Practice, National Health Insurance Corporation Ilsan Hospital, Goyang, Korea; University of Nebraska Medical Center, United States of America

## Abstract

Prehypertension is a risk factor for atherosclerosis. We investigated alterations in plasma metabolites that are associated with prehypertension. A group of 53 individuals was identified who remained within the range of prehypertension during repeated measurements in a 3-year period. This group was compared with the control group of 53 normotensive subjects who were matched for age and gender. Metabolomic profiles were analyzed with UPLC-LTQ-Orbitrap mass spectrometry. The prehypertensive group showed higher levels of lysophosphatidylcholines (lysoPCs) containing C14:0, C16:1, C16:0, C18:2, C18:1, C18:0, C20:5, C20:4, C20:3, and C22:6, higher circulating Lp-PLA_2_ activity, oxidized LDL (ox-LDL), interleukin 6 (IL-6), urinary 8-epi-PGF_2α_, and higher brachial-ankle pulse wave velocity (ba-PWV), before and after adjusting for BMI, WHR, smoking, alcohol consumption, serum lipid profiles, glucose, and insulin. LysoPC (16:0) was the most important plasma metabolite for evaluating the difference between control and prehypertensive groups, with a variable important in the projection (VIP) value of 17.173, and it showed a positive and independent association with DBP and SBP. In the prehypertensive group, the levels of lysoPC (16:0) positively and significantly correlated with ox-LDL, Lp-PLA_2_ activity, 8-epi-PGF_2α_, ba-PWV, and IL-6 before and after adjusting for confounding variables. Prehypertension-associated elevations in lysoPCs, Lp-PLA_2_ activity, ox-LDL, urinary 8-epi-PGF_2α_, IL-6, and ba-PWV could indicate increased oxidative stress from Lp-PLA_2_-catalyzed PC hydrolysis during increased LDL oxidation, thereby enhancing proinflammation and arterial stiffness.

## Introduction

Hypertension is a risk factor for atherosclerosis and cardiovascular disease (CVD) [1]–[4], although the mechanisms by which hypertension is related to atherosclerosis are not clearly established. Several metabolomic studies have been published that investigate the metabolic effects of hypertension [5]–[7]. These studies reported abnormalities in gender-linked steroid patterns [5] or lipid metabolism [6], [7]. Hypertension and its underlying pathophysiology may be present for years before clinical diagnosis, at which time irreversible pathology has already occurred. In 2003, the Seventh Joint National Committee on Prevention, Detection, Evaluation, and Treatment of High Blood Pressure created a prehypertension category for persons with blood pressures ranging from 120–139 mm Hg (systolic) or from 80–89 mm Hg (diastolic). The committee strongly advocated lifestyle and behavioral modifications for individuals with prehypertension [8]. Prehypertension can precede hypertension and atherosclerosis for decades, and it is a condition that represents early CVD. Therefore, it is necessary to determine the role of prehypertension-associated alterations in circulating metabolic profiles. We performed metabolic profiling in a group of 53 individuals who remained within the range of prehypertension during repeated measurements in a 3-year period, and compared these with the metabolic profiles of age- and sex-matched normotensive controls in the same cohort. We also determined lipoprotein-associated phospholipase A_2_ (Lp-PLA_2_) activity, oxidized LDL (ox-LDL), lipid peroxides, and brachial-ankle pulse wave velocities (ba-PWV).

## Materials and Methods

### Study subjects

The 3-year prospective cohort study included 600 healthy subjects (30–65 years old) who underwent triennial medical evaluation from January 2008 to December 2011 through the National Health Insurance Corporation Ilsan Hospital in Goyang, Korea. Prehypertension was defined as blood pressure of 120–139 mm Hg (systolic) and 80–89 mm Hg (diastolic). The screening identified 53 nonobese individuals with normal fasting glucose (29 men and 24 women) who remained within the range for prehypertension during repeated measurements in the 3-year period. From the same population, 53 normotensive control individuals matched for age and sex were recruited who blood pressure has remained within the normal range (systolic blood pressure <120 mm Hg and diastolic blood pressure <80 mm Hg) during repeated measurements in the 3-year period. The purpose of the study was carefully explained to all participants, and written consent was obtained prior to their participation. The Institutional Review Board of the NHIC-sponsored Ilsan Hospital and Yonsei University provided ethical approval of the study protocol, which was performed according to the Helsinki Declaration.

### Anthropometric parameters, blood pressure, blood collection, and dietary intake assessments

Body weight and height of unclothed subjects without shoes were measured in the morning to calculate body mass index (BMI, kg/m^2^). Waist circumference was measured at the umbilical level with the subjects standing after normal expiration. Blood pressure (BP) was measured in the left arm of seated patients with an automatic BP monitor (TM-2654, A&D, Tokyo, Japan) after a 20-minute rest. After a 12-hour fasting period, venous blood specimens were collected in EDTA-treated and plain tubes, centrifuged to produce plasma or serum, and stored at −70°C until analysis. The usual dietary intake of the study subjects was assessed using a semi-quantitative food-frequency questionnaire and a 24-hour recall method [9]. Nutrient intake was determined and calculated based on three-day food records using the Computer-Aided Nutritional Analysis Program (CAN-pro 2.0; Korean Nutrition Society, Seoul, Korea).

### Serum lipid profile and free fatty acids

Fasting levels of total cholesterol and triglyceride were measured using commercially available kits and a Hitachi 7150 Autoanalyzer (Hitachi Ltd., Tokyo, Japan). ApoB-containing lipoproteins were precipitated with dextran-sulfate magnesium, and HDL-cholesterol concentrations in the supernatants were measured enzymatically. For subjects with serum triglyceride levels <400 mg/dL, LDL-cholesterol concentrations were estimated indirectly using the Friedwald formula: LDL-cholesterol  =  Total-cholesterol – [HDL-cholesterol + (Triglycerides/5)]. For subjects with serum triglyceride levels ≥400 mg/dL, LDL-cholesterol concentrations were measured indirectly. Free fatty acids were analyzed with a Hitachi 7150 Autoanalyzer (Hitachi Ltd, Tokyo, Japan).

### Fasting glucose, insulin, and homeostasis-model assessment of insulin resistance

Fasting glucose levels were measured by the glucose-oxidase method with a Beckman Glucose Analyzer (Beckman Instruments, Irvine, CA, USA). Insulin levels were measured by radioimmunoassay using commercial kits from Immuno Nucleo Corporation (Stillwater, MN, USA). Insulin resistance (IR) was calculated by the homeostasis-model assessment (HOMA) using the following equation: HOMA − IR  =  [Fasting insulin (µIU/mL) × Fasting glucose (mmol/L)]/22.5.

### Assessment of serum high-sensitivity C-reactive protein, lipoprotein-associated PLA_2_ activity, plasma malondialdehyde, and LDL particle size

The concentrations of serum high-sensitivity C-reactive protein (hs-CRP) were measured with an Express Plus TM auto-analyzer (Chiron Diagnostics Co., Walpole, MA, USA) using a commercially available, high-sensitivity CRP-Latex(II) X2 kit (Seiken Laboratories Ltd., Tokyo, Japan). The activity of lipoprotein-associated phospholipase A_2_ (Lp-PLA_2_) was measured using a modification of a previously described, high-throughput radiometric activity assay [10]. Plasma malondialdehyde (MDA) was measured from thiobarbituric acid-reactive substances using the TBARS Assay Kit (Zepto-Metrix Co., Buffalo, NY, USA). LDL particles were isolated by sequential flotation ultracentrifugation; the particle size distribution (1.019–1.063 g/mL) was examined by a pore-gradient lipoprotein system (CBS Scientific, CA, USA) on commercially available, non-denaturing, polyacrylamide slab gels containing a linear gradient of 2–16% acrylamide (Alamo Gels Inc., San Antonio, TX, USA). Latex bead (34 nm) conjugated standards of thyroglobulin (17 nm), apoferritin (12.2 nm), and catalase (10.4 nm) were used to estimate the relative migration rates of each band. Gels were scanned using a GS-800 Calibrated Imaging Densitometer (Bio-Rad, Graz, Austria).

### Plasma oxidized LDL, urinary 8-*epi*-prostaglandin F_2α_, serum interleukin-6, interleukin-1β, tumor necrosis factor-α, and measurement of brachial-ankle pulse wave velocity

Plasma ox-LDL was measured using an enzyme immunoassay (Mercodia, Uppsala, Sweden). The resulting color reaction was determined at 450 nm on a Wallac Victor^2^ multilabel counter (Perkin-Elmer Life Sciences, Turku, Finland). Urine was collected in polyethylene bottles containing 1% butylated hydroxytoluene after a 12-hour fast. The bottles were immediately covered with aluminum foil and stored at −70°C until further analysis. The compound 8-*epi*-PGF_2α_ was measured using an enzyme immunoassay (Bioxytech urinary 8-*epi*-PGF_2α_ Assay kit, OXIS International Inc., Portland, OR). Urinary creatinine levels were determined using the alkaline-picrate (Jaffe) reaction. Serum interleukin (IL)-1β, IL-6, and tumor necrosis factor (TNF)–α concentrations were measured using the Bio-Plex Reagent Kit and a Bio-Plex (Bio-Rad Laboratories, Hercules, CA, USA) according to the manufacturer's instructions. Brachial-ankle pulse wave velocity was measured using an automatic waveform analyzer (Model VP-1000; Nippon Colin Ltd., Komaki, Japan) according to a previously published method [11]. The average ba-PWV from both left and right sides was used for analysis.

### Global (nontargeted) metabolic profiling of plasma

#### Sample preparation and analysis

Prior to analysis, 800 µL of 80% acetonitrile was added to 100 µL of plasma, mixed by vortexing, and centrifuged at 10,000 rpm for 5 minutes at 4°C. The supernatant was dried with N_2_, dissolved in 10% methanol, mixed by vortexing, and centrifuged at 10,000 rpm for 5 minutes at 4°C. The supernatant was transferred into a vial.

#### Ultra performance liquid chromatography

The plasma extract samples (7 µL) were injected into an Acquity UPLC-BEH-C18 column (2.1×50 mm, 1.7 µm; Waters, Milford, MA) that was coupled in-line with a UPLC-LTQ-Orbitrap XL (Thermo Fisher Scientific, USA). The injected samples were equilibrated with water containing 0.1% formic acid. Samples were eluted with an acetonitrile gradient containing 0.1% formic acid at a flow rate of 0.35 mL/min for 20 minutes. Metabolites were separated by UPLC (Waters, Milford, MA), analyzed, and assigned by LTQ-Orbitrap-XL (Thermo Fisher Scientific, USA). The mass spectrometer was operated in ESI-positive mode. The spray voltage was 5 kV. The flow-rate nitrogen sheath gas and the auxiliary gas were 50 and 5 (arbitrary units). The capillary voltage (V), tube-lens voltage (V), and capillary temperature (°C) were kept constant at 35 V, 80 V, and 370°C. The Orbitrap data were collected in the range of m/z 50–1,000. For quality control, a mixture of four standard compounds (acetaminophen, sulfadimethoxine, terfenadine, and reserpine) was injected every ten samples. The MS/MS spectra of metabolites were obtained by a collision-energy ramp from 55–65 eV, and conducted with Xcalibur 2.1 and MS Frontier software (Thermo Fisher Scientific, USA).

#### Data processing and identification of metabolites

All MS data including retention times, *m*/*z*, and ion intensities were extracted by SIEVE software (Thermo Fisher Scientific, USA) incorporated into the instrument, and the resulting MS data were assembled into a matrix. SIEVE parameters were set as follows: *m*/*z* range 50–1,000; *m*/*z* width 0.02; retention-time width 2.5; and *m*/*z* tolerance 0.005. Metabolites were searched using the following databases: ChemSpider (www.chemspider.com), Human Metabolome (www.hmdb.ca), Lipid MAPS (www.lipidmaps.org), KEGG (www.genome.jp/kegg), and MassBank (www.massbank.jp). Selected metabolites were confirmed on the basis of retention times and mass spectra of standard samples.

### Statistical analyses

Statistical analyses were performed using SPSS v. 21.0 (IBM SPSS Statistics 21, Chicago, IL, USA). Skewed variables were logarithmically-transformed for statistical analyses. For descriptive purposes, mean values are presented using untransformed values. Results are expressed as means ± standard error (SE). A two-tailed *P*-value of <0.05 was considered statistically significant. Differences in clinical variables between the normotensive and prehypertensive groups at the three-year follow-up were tested using Student's independent *t*-tests. General linear model (GLM) tests were applied to compare changes in variables between the two groups by adjusting for confounding factors. Pearson's and partial correlation coefficients were used to examine the relationships between variables over time. Multiple regression analyses were performed to identify major plasma metabolites of blood pressure. False Discovery Rate (FDR) corrected *q*-values were computed using the R package ‘fdrtool’.

Multivariate statistical analysis was performed using SIMCA-P+ software version 12.0 (Umetrics, Umeå, Sweden). Partial least-squares discriminant analysis (PLS-DA) was used as the classification method for modeling the discrimination between normotensive and prehypertensive subjects by visualizing the score plot (*S*-plot) using the first- and second-PLS components. To validate the model, a seven-fold validation was applied to the PLS-DA model, and the reliabilities of the model were rigorously validated by a permutation test (*n* = 200). The goodness of the fit was quantified by *R* ^2^ *Y*, whereas the predictive ability was quantified by *Q* ^2^ *Y*. Generally, *R* ^2^ *Y* describes how well the data in the training set are mathematically reproduced, and varies between 0 and 1 (a value of 1 indicates a model with a perfect fit). Models with *Q* ^2^ *Y* ≥0.5 are considered to have good predictive capabilities.

## Results

### Clinical characteristics, inflammatory and oxidative-stress markers, and nutrient intake of control and prehypertensive subjects

The mean SBP/DBP levels in the control and prehypertensive groups were 107/65 and 134/84 mm Hg, respectively (Table 1). At baseline, the mean SBP/DBP level in the control group was 105/65 mm Hg, compared with 134/85 mm Hg in the prehypertensive group. Subjects with prehypertension showed significantly higher BMI, waist-hip ratio (WHR), total- and LDL-cholesterol, and triglyceride than controls. The prehypertension group also had higher lipoprotein-associated phospholipase A_2_ (Lp-PLA_2_) activity, plasma malondialdehyde (MDA), urinary excretion of 8-*epi*-PGF_2α_, ba-PWV, and serum interleukin 6 before and after adjusting for BMI, WHR, smoking, alcohol consumption, serum lipid profiles, glucose, and insulin (Table 1). The estimated total caloric intakes were similar in both the prehypertensive group (2,225±40 kcal/d) and the control group (2,194±33 kcal/d). There were no statistically significant differences between the groups with respect to the proportion of caloric intake from macronutrient intake (data not shown), or the intake ratio of polyunsaturated/monounsaturated/saturated (P/M/S) fats between the control (1∶0.80∶0.51) and the prehypertensive (1∶0.80∶0.55) groups. There were no significant differences in the ratio of total energy intake to total energy expenditure between the groups (data not shown).

**Table 1:** Clinical characteristics and inflammatory and oxidative-stress markers.

	Normotensive Group (*n* = 53)	Prehypertensive Group (*n* = 53)	*P*	*P′*
Age (year)	51.1±0.94	51.1±1.30	0.972	−
Male/female (%)	54.7/45.3	54.7/45.3	1.000	−
Systolic BP (mm Hg)	107.4±0.94	134.1±0.76	<0.001	<0.001
Diastolic BP (mm Hg)	64.6±0.94	85.0±0.56	<0.001	<0.001
Body mass index (kg/m^2^)	23.2±0.28	25.3±0.37	<0.001	−
Waist/hip ratio	0.88±0.01	0.91±0.01	0.001	−
Cigarette smoker, *n* (%)	77.4/22.6	86.8/13.2	0.205	−
Alcohol drinker, *n* (%)	30.2/69.8	43.4/56.6	0.159	−
Total-cholesterol (mg/dL)^*^	184.7±5.39	208.2±4.50	<0.001	−
LDL-cholesterol (mg/dL)^*^	116.1±4.60	133.6±3.79	0.001	−
HDL-cholesterol (mg/dL)^*^	51.7±2.05	48.7±1.68	0.277	−
Triglyceride (mg/dL)^*^	89.6±10.4	133.1±12.4	<0.001	−
Glucose (mg/dL)^*^	92.0±1.28	95.9±1.71	0.156	−
Free fatty acid (µEq/L)^*^	468.8±32.0	539.3±28.8	0.050	−
Insulin (µIU/mL)^*^	7.88±0.46	8.92±0.54	0.117	−
HOMA-IR^*^	1.79±0.10	2.14±0.14	0.067	0.783
hs-CRP (mg/dL)^*^	1.15±0.24	1.06±0.23	0.826	0.170
LDL particle size (nm)^*^	24.1±0.15	23.5±0.15	0.005	0.589
Lp-PLA_2_ activity (nmol/mL/min)^*^	28.1±0.81	33.4±0.98	<0.001	0.010
Malondialdehyde (nmol/mL)^*^	9.09±0.33	11.3±0.31	<0.001	0.010
Oxidized LDL (U/L)^*^	38.3±1.30	51.6±2.16	<0.001	0.051
8-*epi*-PGF_2α_ (pg/mg creatinine)^*^	1,111.4±38.8	1,698.2±97.3	<0.001	<0.001
ba-PWV (cm/sec)^*^	2,431.7±44.5	2,991.3±58.4	<0.001	<0.001
Serum IL-6 (pg/mL)^*^	2.00±0.12	3.20±0.14	<0.001	<0.001
Serum IL-1β (pg/mL)^*^	0.61±0.10	0.83±0.16	0.053	0.106
Serum TNF-α (pg/mL)^*^	6.78±0.73	8.55±0.57	0.046	0.100

Means ± S.E.^*^tested by logarithmic transformation. *P*-values derived from independent *t*-tests. *P′*-values derived from independent *t*-tests after adjusting for BMI, WHR, smoking, alcohol consumption, total-cholesterol, LDL-cholesterol, HDL-cholesterol, triglyceride, glucose, free fatty acid, and insulin. HOMA-IR = [fasting insulin (µIU/mL)×fasting glucose (mmol/L)]/22.5. hs-CRP  =  high sensitivity C-reactive protein. ba-PWV = brachial-pulse wave velocity.

### Plasma metabolic profiling using UPLC-LTQ-Orbitrap mass spectrometry

#### Nontargeted metabolic pattern analysis

**Figure 1:**
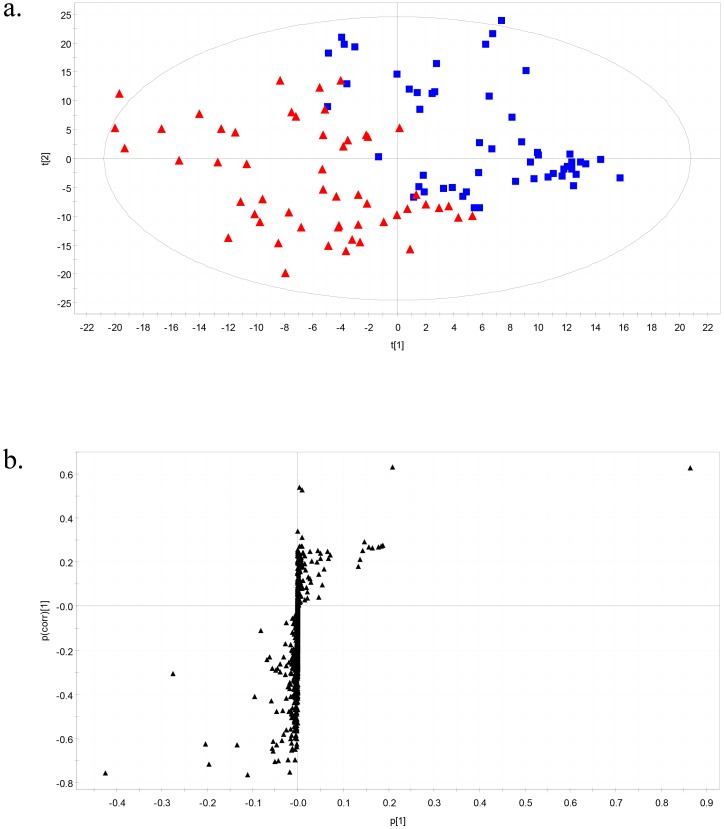
Partial least-squares discriminant analysis models. (**A**) Score plots classifying subjects as normotensive (**filled square**) or prehypertensive (**filled triangle**). (**B**) Score plots for covariance [p] and reliability correlation [p(corr)].

The mass spectrometry (MS) data of plasma metabolites obtained from normotensive and prehypertensive subjects were applied to a PLS-DA score plot (Figure 1a). The two-component PLS-DA score plots of the plasma metabolites showed distinct clustering and clear separation for each of the normotensive and prehypertensive groups. Both groups could be clearly differentiated from each other by the primary component t(1) or the secondary component t(2) based on the model with *R* ^2^ *X*(cum) and *R* ^2^ *Y*(cum) values of 0.232 and 0.661, respectively, which indicates a good fit of the data. The *Q* ^2^ *Y*(cum) value of 0.532 provided an estimate of the predictive ability of the model. The PLS-DA model was validated using a permutation test, which indicated an *R* ^2^ *Y* intercept value of 0.131 and a *Q* ^2^ *Y* intercept value of −0.294. To identify the metabolites that contributed to the differentiation between normotensive and prehypertensive groups, *S*-plots of p(1) and p(corr)(1) were generated using centroid scaling (Figure 1b). The *S*-plot revealed that the metabolites with higher or lower p(corr) values served as the more relevant metabolites for discriminating between the two groups.

#### Identification of plasma metabolites

The metabolites (variables) that play important roles in the differentiation between normotensive and prehypertensive groups were selected according to the variable important in the projection (VIP) parameter (VIP values >1.0 indicate a high relevance to the differences between the sample groups). Among 932 metabolites in plasma, 52 metabolites were selected based on VIP values >1.0; of these, 20 metabolites were identified and 32 were unknown. The results are shown in Table 2. Among the 20 identified plasma metabolites, the normalized peak intensities of 3 amino acids (leucine, phenylalanine, and tryptophan) were significantly higher in prehypertensive subjects than in control subjects; however, these differences disappeared after adjusting for BMI, WHR, smoking, alcohol consumption, serum lipid profiles, glucose, and insulin. Also, these 3 amino acids showed 0.1025, 0.1208, and 0.1000 of *q*-value, respectively. Ten lysophosphatidylcholines (lysoPCs) containing C14:0, C16:1, C16:0, C18:2, C18:1, C18:0, C20:5, C20:4, C20:3, and C22:6 were significantly higher in prehypertensive subjects than in control subjects (*q* = 5.41E-08, 5.05E-14, 6.28E-16, 1.27E-0.5, 4.46E-10, 1.70E-05, 6.81E-05, 6.52E-11, 2.35E-09, and 3.23E-09, respectively) before and after adjusting for all confounding variables including Lp-PLA_2_ activity and ox-LDL.

**Table 2:** Identification of plasma metabolites of normotensive and prehypertensive subjects.

Identity	Formula	Exact Mass	Mass Error (mDa)	Normalized Peak Intensities		Fold Change^a^	*P*	*P′*	*P″*	VIP
	[M + H]+	(M + H)	(mDa)	Normotensive Group (*n* = 53)	Prehypertensive Group (*n* = 53)					
L-Leucine	C_6_H_13_NO_2_	132.1025	−1.3	3,676,275±105,146	3,988,998±96,549	1.09	0.031	0.055	0.248	2.045
L-Phenylalanine	C_9_H_11_NO_2_	166.0868	−1.4	2,247,548±54,466	2,415,093±58,200	1.07	0.038	0.157	0.404	1.079
L-Tryptophan	C_11_H_12_N_2_O_2_	205.0977	−1.3	1,572,767±57,695	1,760,331±62,565	1.12	0.030	0.100	0.187	1.225
Palmitic amide	C_16_H_33_NO	256.2640	−2.1	711,550±97,369	703,228±82,904	0.99	0.948	0.853	0.543	1.156
Oleamide	C_18_H_35_NO	282.2797	−2.2	4,551,082±418,609	4,714,739±407,173	1.04	0.780	0.760	0.621	4.818
LysoPC (14:0)	C_22_H_46_NO_7_P	468.3090	−3.6	261,210±14,207	413,695±18,650	1.58	<0.001	<0.001	<0.001	1.028
LysoPC (16:1)	C_24_H_48_NO_7_P	494.3247	−3.3	551,486±21,985	911,147±30,487	1.65	<0.001	<0.001	<0.001	2.471
LysoPC (16:0)	C_24_H_50_NO_7_P	496.3403	−3.6	9,219,069±155,236	11,815,774±183,606	1.28	<0.001	<0.001	<0.001	17.499
LysoPC (18:2)	C_26_H_50_NO_7_P	520.3403	−4.1	3,670,567±113,737	4,603,096±140,602	1.25	<0.001	<0.001	<0.001	6.010
LysoPC (18:1)	C_26_H_52_NO_7_P	522.3560	−4.6	3,256,978±88,919	4,299,302±103,385	1.32	<0.001	<0.001	<0.001	6.867
LysoPC (18:0)	C_26_H_54_NO_7_P	524.3716	−4.5	4,354,018±129,401	5,339,069±144,196	1.23	<0.001	<0.001	0.001	5.852
LysoPC (20:5)	C_28_H_48_NO_7_P	542.3247	−3.9	437,396±18,626	597,653±28,416	1.37	<0.001	<0.001	0.001	1.088
LysoPC (20:4)	C_28_H_50_NO_7_P	544.3403	−4.0	941,761±23,633	1,285,336±35,484	1.36	<0.001	<0.001	<0.001	2.341
LysoPC (20:3)	C_28_H_52_NO_7_P	546.3560	−4.3	388,869±19,137	592,630±20,649	1.52	<0.001	<0.001	<0.001	1.396
LysoPC (22:6)	C_30_H_50_NO_7_P	568.3403	−4.1	537,886±21,797	807,587±30,720	1.50	<0.001	<0.001	<0.001	1.808
PC (16:0/18:2)	C_42_H_80_NO_8_P	758.5700	−5.6	4,847,743±352,770	4,938,788±384,917	1.02	0.862	0.713	0.602	3.297
PC (16:1/20:4)	C_44_H_78_NO_8_P	780.5543	−5.0	837,185±114,131	835,170±157,967	1.00	0.992	0.964	0.719	1.534
PC (18:2/18:2)	C_44_H_80_NO_8_P	782.5700	−5.1	1,875,687±156,964	2,156,458±157,026	1.15	0.209	0.410	0.528	2.191
PC (18:0/18:2)	C_44_H_84_NO_8_P	786.6013	−5.8	1,200,418±89,200	1,347,258±99,846	1.12	0.275	0.473	0.639	1.791
Lactosylceramide (d18:1/12:0)	C_42_H_79_NO_13_	806.5630	2.6	1,832,933±253,443	1,436,077±185,650	0.78	0.209	0.269	0.548	2.824

Means ± S.E. ^a^Calculated by the mean intensity of each metabolite from the prehypertensive group divided by the mean intensity of each metabolite from the normotensive group. *P*-values derived from independent *t*-tests. *P′*-values derived from independent *t*-tests after adjusting for BMI, WHR, smoking, alcohol consumption, total-cholesterol, LDL-cholesterol, HDL-cholesterol, triglyceride, glucose, free fatty acid, and insulin. *P″*-values derived from independent *t*-tests after adjusting for BMI, WHR, smoking, alcohol consumption, total-cholesterol, LDL-cholesterol, HDL-cholesterol, triglyceride, glucose, free fatty acid, insulin, Lp-PLA_2_, and ox-LDL.

### Blood pressure correlates with clinical and biochemical parameters and major plasma metabolites

In all subjects (*n* = 106), SBP and DBP positively correlated with BMI, WHR, total- and LDL-cholesterol, triglyceride, HOMA-IR index, Lp-PLA_2_ activity, ox-LDL, MDA, 8-*epi*-PGF_2α_, ba-PWV, IL-6, lysoPCs (14:0, 16:1, 16:0, 18:2, 18:1, 18:0, 20:5, 20:4, 20:3, and 22:6) (all *P* values <0.01), and IL-1β (SBP, *P* = 0.040; DBP, *P* = 0.020), and negatively correlated with LDL particle size (all *P* values <0.01). Systolic blood pressure also positively correlated with tryptophan (*P* = 0.015). Based on these results, we performed a multiple-regression analysis to determine independent predictors of SBP and DBP. Age, sex, BMI, WHR, total cholesterol, HOMA-IR index, Lp-PLA_2_ activity, leucine, phenylalanine, tryptophan, and lysoPCs (14:0, 16:1, 16:0, 18:2, 18:1, 18:0, 20:5, 20:4, 20:3, and 22:6) were tested. LysoPC (16:0) emerged as an independent predictor of SBP (standardized *β* = 0.399, *P* = 0.046), as did BMI (standardized *β* = 0.225, *P* = 0.025). LysoPC (16:0) also was an independent predictor of DBP (standardized *β* = 0.453, *P* = 0.025).

### LysoPC (16:0) correlates with ox-LDL, Lp-PLA_2_ activity, 8-*epi*-PGF_2α_, IL-6, IL-1β, TNF-α, and other lysoPCs

**Figure 2:**
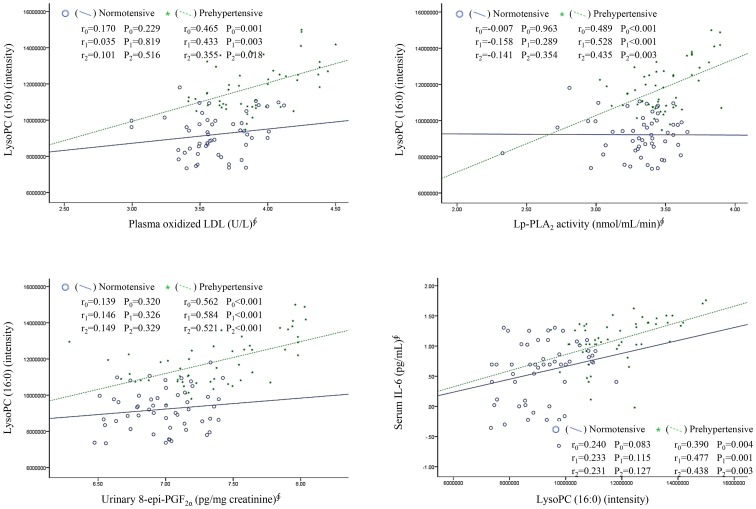
Relationship of lysoPC (16:0) with plasma oxidized LDL, Lp-PLA_2_ activity, urinary 8-*epi*-PGF_2α_, and serum IL-6 in normotensive and prehypertensive subjects. Notes: ^∮^, tested by log-transformed; *r_0_*, tested by Pearson correlation analysis (unadjusted); *r_1_*, tested by partial correlation analysis (after adjusting for age, sex, BMI, WHR, smoking, and alcohol consumption); *r_2_*, tested by partial correlation analysis (after adjusting for age, sex, BMI, WHR, smoking, alcohol consumption, and LDL-cholesterol).

In prehypertensive subjects, the levels of lysoPC (16:0) positively and significantly correlated with ox-LDL, Lp-PLA_2_ activity, 8-*epi*-PGF_2α_, and IL-6 before and after adjusting for BMI, WHR, smoking, alcohol consumption, and LDL cholesterol (Figure 2). However, these associations were not found in normotensive subjects. In both prehypertensive and control subjects, the levels of lysoPC (16:0) positively correlated with other lysoPCs (14:0, 16:1, 18:2, 18:1, 18:0, 20:5, 20:4, 20:3, and 22:6) and ba-PWV (all *P* values <0.014). Serum IL-6, IL-1β, and TNF-α positively correlated with urinary 8-*epi*-PGF_2α_ (*r* = 0.262, *P* = 0.007; *r* = 0.281, *P* = 0.042; *r* = 0.373, *P* = 0.006, respectively) and plasma MDA (*r* = 0.449, *P*<0.001; *r* = 0.279, *P = *0.043; *r* = 0.277, *P = *0.044, respectively).

## Discussion

This study identified prehypertension-associated alterations in lysoPC levels and amino acid metabolism. Significant differences in the metabolic profiles were found between prehypertensive and normotensive individuals, including lysoPCs (14:0, 16:1, 16:0, 18:2, 18:1, 18:0, 20:5, 20:4, 20:3, and 22:6) and amino acids (leucine, phenylalanine, and tryptophan). Among the 10 lysoPCs, the majority contained long-chain acyl groups (i.e., C≥16) [12]. This result is in agreement with previous studies on the effects of long-chain lysoPC (C≥16) in vasodilation impairment [13], and the identification of higher levels of lysoPCs with long-chain acyl groups in the plasma of spontaneously hypertensive rats.^6^ This study identified lysoPC (16:0) (VIP value of 17.173) as the most important plasma metabolite for evaluating the difference between prehypertensive and normotensive individuals. LysoPC (16:0) was positively and independently associated with DBP and SBP. This observation could support previous work on the role of increased lysoPC (16:0) in arterial stiffness [14].

LysoPC constitutes only 1–5% of the total PC content of non-ox-LDL; however, as much as 40–50% of the PC contained within LDL is converted to lysoPC during LDL oxidation [15]. A saturated or monounsaturated fatty acid predominates in the *sn*-1 position of the phospholipid [16]. The generation of free radicals as a result of oxidative stress can activate PLA_2_, which catalyzes the hydrolysis of the ester bond at the *sn*-2 position of phospholipids. Stafforini et al. [16] showed that the secreted form of Lp-PLA_2_ released F_2_-isoprostanes (the end-products of lipid oxidation) from the *sn*-2 position of PC with high affinity. Kono et al. [17] reported that intracellular type II Lp-PLA_2_, which shares homology with the plasma enzyme Lp-PLA_2_, was involved in the metabolism of esterified 8-*iso*-PGF_2α_. We also measured prehypertension-associated elevations in plasma Lp-PLA_2_ activity, plasma ox-LDL, lysoPCs, and urinary 8-*epi*-PGF_2α_. These results are in agreement with a previous report of higher mean levels of Lp-PLA_2_ and 8-*epi*-PGF_2α_ in subjects with high ox-LDL than those in subjects with low ox-LDL [18], and a positive association between plasma ox-LDL and Lp-PLA_2_ activity in metabolic syndrome [19].

In prehypertensive individuals, a strongly positive correlation of lysoPC (16:0) with ox-LDL, Lp-PLA_2_ activity, and 8-*epi*-PGF_2α_ (a sensitive marker for oxidative stress) [20], [21] could indicate increased production of oxidative stress from Lp-PLA_2_-catalyzed PC hydrolysis during increased LDL oxidation. This result is consistent with a previous report of a correlation between increased Lp-PLA_2_ activity and increased levels of lysoPC, ox-LDL, and cytokine in hypercholesterolemic minipigs [22]. A close correlation between the circulating lysoPC (16:0) levels and urinary 8-*epi*-PGF_2α_ also was observed in middle-aged nonobese men [14]. However, this association was not found in normotensive subjects, possibly due to low levels of LDL cholesterol, ox-LDL, and Lp-PLA_2_ activity.

LysoPC, a major atherogenic component of oxLDL, was elevated in the plasma of atherosclerotic patients [23], and was an important biomarker of coronary artery disease (CAD) [24]. Lp-PLA_2_ levels were observed to correlate with three lysoPCs (16:0, 18:0, and 18:1) in carotid plaques before and after adjusting for age, sex, hypertension, CAD, and statin usage [10]. A close association between circulating lysoPC (16:0) and the ba-PWV index of central arterial stiffness [25], [26], and serum IL-6, was reported in human subjects [14]. Production of lysoPC (16:0) can stimulate the expression of adhesion molecules and the release of cytokines in endothelial cells [26]. IL-6 was induced by lysoPC (16:0) treatment in human umbilical vein endothelial cells. LysoPC (16:0) induced higher release of arachidonic acid mediated via cytosolic PLA_2_ from human coronary artery smooth muscle cells, compared to that for treatment with lysoPC (14:0) or unsaturated lysoPC [27]. LysoPC has been also shown enhance the release of IL-6 from peripheral blood mononuclear cells [28], regulated on activation normal T-cell expressed and secreted from microvascular endothelial cells [29], and TNF-α [30] as well as IL-1β from human monocytes [31]. In addition, previous epidemiologic studies have linked higher plasma concentrations of inflammatory markers - IL-6 [32], [33], IL-1β [34], and TNF-α [35] - to increased SBP and DBP or hypertensive status. Consistent with these reports, the current study showed significant increases in ba-PWV, serum IL-6 levels, and lipid peroxides including 8-*epi*-PGF_2α_ and MDA in the prehypertensive group, and positive correlations among lysoPC (16:0), IL-6, 8-*epi*-PGF_2α_, and MDA.

LysoPCs represent 5–20% of the total plasma phospholipids, and are formed by the action of lecithin cholesterol acyltransferase (LCAT) in plasma [36]. Human LCAT releases lysoPC 20:4 and 22:6 from the *sn*-1 position of PC [37]. Up to 80% of the lysoPC in plasma is found in the non-lipoprotein fraction, in which albumin is considered as the main lipid-binding protein [36], [38]. Unsaturated lysoPCs are associated primarily with albumin rather than lipoproteins. We observed a positive relationship between the levels of lysoPC (16:0) and those of other lysoPCs (14:0, 16:1, 18:2, 18:1, 18:0, 20:5, 20:4, 20:3, and 22:6), which could reflect an alternative source of lysoPC (16:0) production in addition to ox-LDL. LysoPC is formed by PLA_2_-induced hydrolysis or oxidation of PC in LDL and cell membranes [23]. LysoPCs (14:0, 16:0, 18:0, and 18:1) and branched-chain or aromatic amino acids were elevated in overweight/obese, insulin-resistant subjects compared with the levels in lean control subjects [39]-[42]. In the current study, differences in the levels of leucine, phenylalanine, and tryptophan between control and prehypertensive subjects disappeared after adjusting for BMI, WHR, smoking, alcohol consumption, serum lipid profiles, glucose, and insulin. However, differences in the levels of lysoPCs in the two groups remain highly significant. This result suggests an important role of lysoPCs in prehypertension, independently from confounding variables.

Our results have similar limitations as all cross-sectional and observational studies. We evaluated associations rather than prospective prediction; thus, the causal relationships among the identified metabolites and the exact mechanisms of the prehypertensive changes are unknown. A large number of markers were detected using UPLC-LTQ-Orbitrap MS, but most remain unidentified. Unlike the large databases for GC-MS, the endogenous biomolecule databases for LC-MS-based metabolomics research have not yet been constructed [43]. Our study used UPLC-LTQ-Orbitrap MS-based metabolomics and multivariate data analyses to identify a cluster of prehypertension-associated changes in plasma metabolites for lysoPCs containing C14:0, C16:1, C16:0, C18:2, C18:1, C18:0, C20:5, C20:4, C20:3, and C22:6, which were significant before and after adjusting for BMI, WHR, smoking, alcohol consumption, serum lipid profiles, glucose, and insulin. LysoPC (16:0) had a VIP value of 17.173 and showed positive and independent association with DBP and SBP. We also found prehypertension-associated elevations in Lp-PLA_2_ activity, ox-LDL, urinary 8-*epi*-PGF_2α_, IL-6, and ba-PWV. These results could indicate increased production of lysoPCs and oxidative stress from Lp-PLA_2_-catalyzed PC hydrolysis during increased LDL oxidation in prehypertension. The potential activation of a proinflammatory phenotype could result in arterial stiffness related to prehypertension, a risk factor for atherosclerosis [44].

## Conclusions

In summary, the results of our metabolomics analysis of plasma from normotensive versus prehypertensive subjects offer novel insights into metabolic alterations occurring during the prehypertensive period preceding the manifestation of hypertension or atherosclerosis. These results could provide valuable candidates for new intervention targets.
